# A Study on the Self-Reported Physician-Diagnosed Cardiac Complications Post mRNA Vaccination in Saudi Arabia

**DOI:** 10.7759/cureus.52108

**Published:** 2024-01-11

**Authors:** Muazzam M Sheriff, Renad Ahmed A Marghalani, Ohoud Mohammed M Almana, Wardah Mohammed Yousef A Almaimani, Yasmin Fahad A Saidi, Sahar Fawzi S Arbaeen, Atheer K Almutairi, Atheer G Alharbi, Ranya M Aljehani, Hesham Mortadh M Alhasan

**Affiliations:** 1 Microbiology and Immunology, Ibn Sina National College for Medical Studies, Jeddah, SAU; 2 Medicine and Surgery, Ibn Sina National College for Medical Studies, Jeddah, SAU; 3 Medicine and Surgery, Ibn Sina National College For Medical Studies, Jeddah, SAU; 4 Critical Care, King Faisal General Hospital, Makkah, SAU

**Keywords:** post-vaccination, diagnosed, complications, cross-section study, mrna

## Abstract

Introduction

The advent of mRNA-based vaccines has been a pivotal milestone in the global response to the pandemic, prompting widespread vaccination campaigns, including in Saudi Arabia. This study investigates self-reported physician-diagnosed cardiac complications post mRNA vaccination in Saudi Arabia, recognizing the need to monitor these rare events. The research aimed to study the self-reported physician-diagnosed incidence, nature, and associated factors of cardiac complications in this distinctive demographic group of post-mRNA vaccinations. Due to the scarcity of specific vaccine safety data, the study sought to provide data supporting public awareness and recommendations for global public health practices. Emphasizing ethical considerations, the study focuses on local factors, contributing valuable insights into the safety profile of mRNA vaccines, and aiding informed decision-making for public health strategies in Saudi Arabia and globally.

Material and methods

In a cross-sectional design, the study employs a culturally adapted questionnaire covering demographic details, vaccination history, health conditions, and perceptions. A rigorous development and validation process ensured the reliability of the questionnaire. A sample size of 804 participants was collected through an online survey link distributed via social media through the groups. Statistical analyses explored associations between demographic variables, vaccination behaviors, health diagnoses, and perceptions using IBM SPSS version 23 and Microsoft Excel.

Results

Significant associations were found among demographic variables, vaccination behaviors, health diagnoses, and perceptions of self-reported physician-diagnosed cardiac complications post mRNA vaccination in Saudi Arabia. Key findings included a high mRNA vaccine uptake with a frequency of 747 (92.79%) and a mere frequency of 218 (27.11%) reporting cardiac complications post vaccination. The study highlighted diverse influences on vaccine decisions, with a frequency of 384 (47.76%) expressing neutral confidence in vaccine safety. The study contributes to the global understanding of mRNA vaccine safety, emphasizing the unique Saudi demographic context. Methodological rigor, ethical considerations, and acknowledgment of limitations enhance credibility. Collaborative efforts and tailored recommendations for public health policies and communication strategies are underscored.

Conclusion

This study on self-reported physician-diagnosed cardiac complications post mRNA vaccination in Saudi Arabia is a crucial addition to global vaccine safety research. Providing insights shaped by local factors, the study aids in informed decision-making for public health strategies in Saudi Arabia and globally. It addresses the scarcity of specific vaccine safety data, fostering a nuanced understanding of mRNA vaccine-related cardiac complications worldwide.

## Introduction

The advent of mRNA-based vaccines stands as a pivotal achievement in the global battle against the pandemic. As numerous nations embarked on extensive vaccination campaigns to curb virus transmission, the imperative to monitor and comprehend potential side effects, especially those impacting the cardiovascular system, became apparent [1]. Though cardiac complications following vaccination are rare, global reports have necessitated a thorough investigation into the occurrence and characteristics of self-reported physician-diagnosed cardiac complications among those who have received mRNA vaccines in Saudi Arabia, as it boasts a distinctive demographic and epidemiological profile, necessitating an understanding of how mRNA vaccination affects cardiac health within this context [2]. Due to the scarcity of data on vaccine safety specific to the Saudi population, a dedicated study becomes imperative to uncover the incidence, nature, and associated factors of self-reported physician-diagnosed cardiac complications post mRNA vaccination [3].

Cardiac complications, albeit infrequent, carry significant implications for public health. Grasping and comprehending these complications after mRNA vaccination is vital for a comprehensive assessment of vaccine safety and for guiding public health measures [4]. Continuous monitoring of vaccine safety is paramount, and investigating self-reported physician-diagnosed cardiac complications post mRNA vaccination provides real-world data to complement clinical trial findings, enhancing our understanding of the long-term safety profile of these vaccines [5]. By scrutinizing self-reported cases, the study aims to raise awareness of potential cardiac complications among the general population and healthcare providers, fostering early detection, prompt reporting, and appropriate management [6]. Identifying specific risk factors or patterns of cardiac complications post mRNA vaccination can optimize healthcare resources, including targeted monitoring, efficient facility utilization, and streamlined healthcare responses, ultimately benefiting both patients and healthcare systems [7]. Addressing concerns related to cardiac complications following mRNA vaccination contributes to bolstering public confidence in vaccination programs, where accurate information about risks and benefits is essential for maintaining high vaccination rates and achieving population immunity [8].

The unique genetic, cultural, and lifestyle factors in the Saudi population may influence responses to mRNA vaccines [9]. Investigating cardiac complications within this specific context will provide insights directly applicable to the local population, enabling tailored public health interventions [10]. As vaccine hesitancy remains a global concern, especially with emerging vaccine technologies, transparently communicating and investigating cardiac complications post mRNA vaccination is crucial for maintaining and building public trust [11]. Providing accurate information can alleviate concerns and enhance confidence in the vaccination program [12].

The study findings can inform public health policies and guidelines related to mRNA vaccination. If specific risk factors or patterns emerge, targeted recommendations can be developed to maximize vaccination benefits while minimizing potential risks [13]. The clinical trials provide valuable insights, and real-world evidence is equally important for understanding how vaccines perform in diverse populations and under everyday conditions [14]. This study contributes real-world data, complementing clinical trial findings and providing a more comprehensive understanding of vaccine safety in this context.

The ethical imperative ensures the well-being of vaccine recipients; investigating self-reported cardiac complications is not only scientifically justified but also ethically responsible. The study contributes to the ongoing commitment to prioritize safety in vaccine administration. Adding to the growing body of global knowledge on the safety profile of mRNA vaccines, cross-referencing findings from different regions enhances our understanding of potential cardiac complications, contributing to a more comprehensive and nuanced assessment of the safety of mRNA vaccines worldwide [15].

This cross-sectional study included determining the frequency, characterizing the nature and severity, investigating the temporal relationship between vaccination and cardiac complications, identifying associated risk factors, and evaluating healthcare-seeking behavior for cardiac complications [16]. To achieve these aims, the study outlined specific objectives. It aims to conduct a comprehensive cross-sectional survey, developed and administered to individuals who have received mRNA vaccines in Saudi Arabia. Additionally, the study intends to review medical information and obtain consent to validate and corroborate self-reported cardiac complications. Incidence rates will be calculated, stratifying data by demographic and clinical variables, and cardiac complications will be characterized in terms of nature and severity, considering symptomatology, diagnostic procedures, and clinical outcomes [17]. The temporal relationship between mRNA vaccination and the onset of cardiac complications will be analyzed to explore patterns or clusters of cases [18]. Statistical analyses will be employed to identify potential risk factors associated with the occurrence of self-reported physician-diagnosed cardiac complications post mRNA vaccination, considering variables such as age, gender, comorbidities, and vaccination specifics [19]. The study also explored healthcare-seeking behavior and examined patterns among individuals reporting cardiac complications, including the types of healthcare facilities visited and the timeliness of seeking medical attention. Furthermore, the findings are summarized to provide recommendations for public health practices, vaccination guidelines, and communication strategies [20]. Finally, the study aimed to contribute to the global understanding of mRNA vaccine safety by disseminating research findings, facilitating comparisons with international data, and enriching the collective knowledge base on vaccine-related cardiac complications. By accomplishing these aims and objectives, the study endeavors to offer valuable insights into the safety profile of mRNA vaccines in the population. The timely and critical nature of this research was vital for ensuring the ongoing success and safety of the vaccination campaign and providing value to the population in a global context.

The primary aims of this study are to assess the incidence of self-reported physician-diagnosed cardiac complications among individuals who have received mRNA vaccines in Saudi Arabia. A study on self-reported physician-diagnosed cardiac complications post mRNA vaccination in Saudi Arabia was not only timely but also critical for ensuring the success and safety of future vaccination campaigns. This research will contribute valuable data to the global understanding of mRNA vaccine safety and provide insights specific to the unique characteristics of the studied population.

## Materials and methods

Study design

This investigation employed a cross-sectional research design to explore the dynamics of evaluating self-reported physician-diagnosed cardiac complications following mRNA vaccination in Saudi Arabia. The research methodology involved developing and validating a comprehensive questionnaire, collecting data, and subsequently analyzing it [21].

Questionnaire development

A detailed questionnaire was created to assess self-reported physician-diagnosed cardiac complications post mRNA vaccination in Saudi Arabia. The questionnaire encompassed sections on demographic details, knowledge and awareness-based inquiries, psychosocial aspects, frequency of cases, hesitancy, vaccination history, self-reported cardiac symptoms, healthcare-seeking behavior, relevant medical history, and validated assessment scales of patient health [22].

Translation and cultural adaptation

To ensure cultural relevance and linguistic accuracy, a meticulous translation and adaptation process was undertaken for the questionnaire. Certified licensed bilingual experts translated the English version into Arabic, followed by a separate back-translation by a different set of certified licensed bilingual experts. The consensus resolution addressed any discrepancies, preserving the original intent while aligning with the local cultural context [23].

Questionnaire validation

The translated questionnaire underwent validation to ensure its reliability and validity. The Cronbach’s alpha was 0.77344 for the questionnaires. Content validation involved a panel of experts, including a critical care expert. A pilot study on a small sample size of 10 participants assessed the clarity and comprehensibility of questionnaire items, with participant feedback used to refine them [24]. The pilot study participants were also included in the actual study, and the confidentiality of their data, including demographic information, was preserved as part of ethical consideration.

Sample size

The study's sample size was determined using the Raosoft online sample calculator (Raosoft Inc., Seattle, WA), targeting a 95% confidence level with a margin of error within ±5%. The decision to select a sample size of 804 participants for the cross-sectional study on self-reported physician-diagnosed cardiac complications post mRNA vaccination in Saudi Arabia was influenced by several crucial factors, including the anticipated variability in the population regarding cardiac complications, the desired confidence level and margin of error for accurate estimates, and the statistical power needed to detect significant effects. Additionally, the study's design, the complexity of the variables involved, and the planned analyses played a role in determining the optimal sample size. Practical considerations, including available resources and potential constraints, have also influenced this decision. The chosen sample size reflected a careful balance between statistical precision and feasibility, aiming to provide meaningful insights into the incidence and nature of cardiac complications in the specific demographic context of Saudi Arabia. The confidence level represents the certainty with which conclusions about the population are based on the sample, as the common choices are 95% or 99%. A higher confidence level provided a larger sample size. The calculated sample size required was 804, based on inclusion criteria set by a specialist critical care professional [25].

Inclusion and exclusion criteria

The inclusion criteria for the study encompass individuals aged 18 and above, residents of Saudi Arabia, and those who have received at least one dose of an mRNA vaccine (Pfizer-BioNTech, Moderna, or both). Voluntary participation with informed consent was mandatory to uphold ethical standards. The consent was mentioned on the first page of the survey form and will proceed to the questionnaires only upon acceptance. The exclusion criteria include individuals below the age of 18, those who have not received any mRNA vaccine dose, individuals unable to provide informed consent by acceptance on the first page of the survey form, non-residents of Saudi Arabia, as the study was only intended for the residents of urban areas to justify the study population mentioned in the title, and those providing inconsistent or unreliable survey responses. Maintaining data accuracy and ensuring the study's focus on the local population were key considerations in determining eligibility. These criteria collectively aimed to yield meaningful insights into the incidence and nature of cardiac complications post mRNA vaccination within the specific demographic context of Saudi Arabia.

Ethical approval

The research adhered rigorously to ethical protocols, prioritizing participant anonymity and privacy and obtaining informed consent. Approval was granted by the Ibn Sina National College (ISNC) Institutional Research Review Board (IRRB), Jeddah, Saudi Arabia, under the ethical approval reference number IRRB-03-15102023.

Data collection

Informed consent was obtained, emphasizing confidentiality and voluntary participation. The study was conducted from September 17, 2023, to November 16, 2023, with 804 participating volunteers aged 18 and above from diverse geographic regions to ensure a representative sample. The study was conducted on individuals who had previously experienced the onset and duration of cardiac complications diagnosed by a physician post-vaccination, which varied from less than a month to 12 months. The online survey created with a Google Form (Google Inc., Mountainview, CA) in English was used for the cross-sectional study [26]. A non-probability-purposed sampling technique was employed for participant selection. The distribution strategy involved sharing the survey with the intended population through group posts and direct messages, ensuring broad accessibility for potential participants [27]. Before participation, individuals were provided with informed consent, highlighting their commitment to maintaining confidentiality and emphasizing the voluntary nature of their involvement.

Data analysis

A thorough statistical analysis was conducted on the collected data. Descriptive statistics presented demographic and intricacy-related variables describing the complications. Validated assessment scales were analyzed to quantify intricacy prevalence and severity [25]. Inferential statistics, including chi-square tests and regression analysis, explored relationships between intricacy and various factors. IBM SPSS software version 23 (IBM Corp., Armonk, NY) and Microsoft Excel (Microsoft Corp., Redmond, WA) were employed for data analysis, maintaining a statistical power of 80% at a cutoff value of 0.05 [24].

## Results

A study on self-reported physician-diagnosed cardiac complications following mRNA vaccination in Saudi Arabia revealed significant associations between demographic variables, vaccination behaviors, health diagnoses, and perceptions of participants who reported physician-diagnosed cardiac complications after the administration of mRNA vaccination in Saudi Arabia. The findings provide a nuanced understanding of the diverse factors influencing vaccine outcomes and perceptions within the surveyed population. These results can guide public health interventions, communication strategies, and targeted healthcare approaches to consider the specific concerns and health outcomes associated with mRNA vaccination. Further research and monitoring are essential for the continuous assessment and adaptation of strategies in response to evolving patterns and concerns.

Demographic variables

The survey included 804 participants from various regions, with the Western region exhibiting the highest response rate at 26.12%. The gender distribution was nearly balanced, and participants spanned diverse age groups, nationalities, marital statuses, and educational backgrounds. Statistically significant differences (p ≤0.05) were achieved using Pearson's chi-square test from the responses based on province, gender, age group, nationality, marital status, and education. The details of the demographic variables are mentioned in Table 1.

**Table 1:** Demographic characteristics of the study group

Survey questionnaires	Response rate: absolute number (total=804)	Response rate: percentage	p-value (p ≤0.05)
Province			0.042
Northern	127	15.80%	
Southern	166	20.65%	
Eastern	174	21.64%	
Western	210	26.12%	
Central	127	15.8%	
Gender			0.051
Male	379	47.14%	
Female	425	52.86%	
Age group (in years)			0.043
18-24	194	24.13%	
25-34	221	27.49%	
35-44	175	21.77%	
45-54	167	20.77%	
55 and above	47	5.85%	
Nationality			0.037
Saudi	543	67.41%	
Non-Saudi	261	32.49%	
Marital status			0.029
Single	179	22.26%	
Married	625	77.74%	
Education			0.039
Undergraduate	254	31.59%	
Graduate	371	46.14%	
Postgraduate	79	9.83%	
Doctorate	17	2.11%	
Others	83	10.32%	

Vaccination information

A majority of participants (92.79%) reported receiving mRNA vaccines, with Pfizer-BioNTech (43.18%) and Moderna (27.49%) being the predominant choices. The number of vaccine doses received varied, with one dose being the most common (39.43%). Statistically significant differences (p ≤0.05) were observed in responses related to vaccine uptake and types. The details of the vaccination information are mentioned in Table 2.

**Table 2:** Information about vaccination received by the study group

Survey questionnaires	Response rate: absolute number (total=804)	Response rate: percentage	p-value (≤0.05)
Have you received an mRNA vaccine?			0.011
Yes	747	92.79%	
No	57	7.21%	
Specify which mRNA vaccine(s) you have received.			0.045
Pfizer-BioNTech	347	43.18%	
Moderna	221	27.49%	
Both Pfizer-BioNTech and Moderna	178	22.14%	
None	0	0%	
Other	58	7.21%	
How many doses of the mRNA vaccine have you received?			0.042
One	317	39.43%	
Two	297	36.95%	
Three	120	14.93%	
Four	70	8.71%	

Health conditions before vaccination

Pre-existing health conditions were reported by participants, with diabetes (48.26%) and hypertension (56.72%) being the most prevalent. Statistically significant differences (p ≤0.05) were noted in the prevalence of various health conditions, reflecting the diversity of the sample. The details of the health conditions before vaccination are mentioned in Table 3.

**Table 3:** The details of the study group's health conditions before vaccination

Survey questionnaires	Response rate: absolute number (total=804)	Response rate: percentage	p-value (p ≤0.05)
Have you been diagnosed with diabetes mellitus?			0.049
Yes	387	48.26%	
No	417	51.74%	
Have you been diagnosed with hypertension?			0.046
Yes	456	56.72%	
No	348	43.28%	
Have you been diagnosed with dyslipidemia?			0.027
Yes	173	21.52%	
No	631	78.48%	
Have you been diagnosed with obesity?			0.039
Yes	315	39.15%	
No	489	60.85%	
Have you been diagnosed with any health issues that may be related to your sedentary lifestyle?			0.028
Yes	178	22.14%	
No	626	77.86%	
Have you been diagnosed with any smoking-related health issues?			0.027
Yes	221	27.49%	
No	583	72.51%	

Health outcomes following mRNA vaccination

Approximately 27.11% of participants reported being diagnosed with cardiac complications post mRNA vaccination. Duration, hospitalization, and treatment methods varied significantly, with medical treatment being the most common. The onset of cardiac complications was reported within different timeframes. Statistically significant differences (p ≤0.05) were observed in responses related to cardiac complications, hospitalization, and treatment. The details of the health outcomes after vaccination are mentioned in Table 4.

**Table 4:** The study group's health outcomes post vaccination

Survey questionnaires	Response rate: absolute number (Total=804)	Response rate: percentage	p-value (p ≤0.05)
Have you been diagnosed with cardiac complications by a physician?			0.024
Yes	218	27.11%	
No	586	72.89%	
What is the onset duration of cardiac complications diagnosed by a physician post vaccination?			0.032
Less than 1 month	117	14.55%	
1-3 months	53	6.97%	
3-6 months	31	3.86%	
6-12 months	11	1.37%	
More than 12 months	6	0.75%	
Not applicable	586	45.77%	
Did you get admitted to the hospital?			0.023
Yes	218	27.11%	
No	586	72.89%	
Have you been admitted to the critical care unit or ward?			0.025
Critical care unit	127	15.80%	
Ward	91	11.44%	
No	586	72.89%	
What was the duration of hospitalization?			0.024
Less than 1 day	56	6.97%	
1-3 days	89	11.07%	
4-7 days	67	8.33%	
1-2 weeks	4	0.50%	
2-4 weeks	2	0.25%	
More than 4 weeks	0	0%	
Not applicable	586	72.89%	
What is the duration of treatment?			0.023
Less than 1 month	7	0.87%	
1-3 months	13	1.62%	
3-6 months	11	1.37%	
6-12 months	53	6.59%	
More than 12 months	76	9.45%	
Continuous	57	7.11%	
No current treatment	1	0.12%	
Not applicable	586	72.89%	
What is the treatment method?			0.022
Medical	197	24.50%	
Procedure	21	2.61%	
Not applicable	586	72.89%	

Perceptions and concerns

Participants expressed varying levels of confidence in the safety of mRNA vaccines, with 47.76% being neutral. A notable proportion believed that their cardiac complications were related to the vaccine. Influences on vaccine decisions included healthcare professionals (22.89%) and government health agencies (34.45%). Statistically significant differences (p ≤0.05) were observed in responses related to vaccine confidence, perceived vaccine-cardiac complications relationship, and sources of information. The details of the perceptions and concerns are mentioned in Table 5.

**Table 5:** The participants' perceptions and concerns regarding the mRNA vaccine

Survey questionnaires	Response rate: absolute number (total=804)	Response rate: percentage	p-value (p ≤0.05)
How confident are you in the safety of the mRNA vaccine?			0.044
Very confident	127	15.80%	
Somewhat confident	156	19.40%	
Neutral	384	47.76%	
Somewhat not confident	67	8.33%	
Not confident at all	70	8.71%	
To what extent do you believe that the cardiac complications you experienced were related to the mRNA vaccine?			0.042
Strongly related	73	9.08%	
Somewhat related	84	10.45%	
Neutral	366	45.52%	
Not very related	137	17.01%	
Not related at all	144	17.91%	
What sources of information influenced your decision to receive the mRNA vaccine?			0.040
Healthcare professionals	184	22.89%	
Government health agencies	277	34.45%	
Scientific studies and research	16	1.99%	
Family and friends	79	9.85%	
News media	103	12.81%	
Social media	139	17.27%	
Other	6	0.75%	

The analysis conducted on the survey questionnaires unveiled significant relationships between various demographic variables and participants' responses, as denoted by p-values (p ≤0.05). In Table 1, demographic data indicates noteworthy findings, such as the Western region exhibiting the highest response rate at 26.12%, a balanced gender distribution, and diverse educational levels. Table 2 delves into vaccination information, revealing a high uptake of mRNA vaccines (92.79%) and significant preferences for Pfizer-BioNTech and Moderna. Table 3 explores health conditions before vaccination, highlighting prevalent diagnoses such as diabetes mellitus (48.26%) and hypertension (56.72%). Table 4 examines health outcomes post-vaccination, with 27.11% reporting cardiac complications and diverse treatment durations. Table 5 focuses on perceptions and concerns, revealing varied confidence levels in vaccine safety and influences from healthcare professionals and government health agencies. These regression findings provide comprehensive insights into the intricate relationships between demographic factors, vaccination behaviors, health diagnoses, and perceptions, offering valuable data for targeted public health strategies. These regression findings provide valuable insights into the relationships between demographic variables, vaccination behaviors, health diagnoses, and perceptions. The statistical significance of these associations contributes to a nuanced understanding of the survey responses. Future research may delve deeper into specific demographic factors or health outcomes to inform targeted interventions and public health strategies.

## Discussion

The study on self-reported physician-diagnosed cardiac complications post mRNA vaccination in Saudi Arabia represents a pivotal contribution to the ongoing global efforts to understand the safety profile of mRNA-based vaccines. This research emerges against the backdrop of the unprecedented development and deployment of mRNA vaccines in response to the pandemic [28]. As Saudi Arabia, like many nations, vigorously rolls out vaccination campaigns to curb virus transmission, the imperative to closely monitor and comprehend potential side effects, particularly those affecting the cardiovascular system, becomes increasingly apparent [4].

This study acutely recognizes the rarity of cardiac complications post-vaccination, as reported globally [3]. However, the recognition of these rare events necessitates a thorough investigation into the incidence, nature, and associated factors of self-reported physician-diagnosed cardiac complications among those who have received mRNA vaccines within the unique demographic and epidemiological landscape of Saudi Arabia [29].

This study acknowledges the unique genetic, cultural, and lifestyle factors inherent in the Saudi population. These factors may play a crucial role in influencing how individuals respond to mRNA vaccines [7]. By delving into cardiac complications within this specific context, the study not only provides localized insights but also lays the foundation for tailoring public health interventions that resonate with the Saudi population [10].

The meticulous approach to questionnaire development, translation, and cultural adaptation ensures the relevance and accuracy of the data collected [23]. The emphasis on ethical considerations underscores the commitment to the well-being of vaccine recipients, aligning with the broader ethical imperatives of scientific research [22]. The study’s comprehensive objectives, ranging from assessing incidence to evaluating healthcare-seeking behavior, highlight a dedication to generating a nuanced understanding of the impact of mRNA vaccination on cardiac health.

By providing valuable insights, future longitudinal studies could offer a deeper understanding of the evolution of cardiac complications over time. The transparent acknowledgment of limitations, coupled with the robust validation process of the questionnaire, contributes to the study's credibility.

In comparing this study with similar research endeavors globally, common concerns surrounding cardiac complications post mRNA vaccination are evident [30]. The emphasis on collaborative efforts, global knowledge enrichment, and the dissemination of findings for international comparisons aligns with the collective endeavor to enhance the understanding of vaccine-related complications on a global scale [30].

The study's findings carry substantial implications for public health policies globally. The tailored recommendations informed by the study could prove instrumental in maximizing the benefits of vaccination programs while minimizing potential risks [9]. The communication strategies outlined in the study contribute to the broader global challenge of building and maintaining public trust in vaccination, especially with emerging vaccine technologies [20].

The study on self-reported physician-diagnosed cardiac complications post mRNA vaccination in Saudi Arabia stands as a comprehensive and timely contribution to the evolving landscape of vaccine safety research [22]. Its methodological rigor, cultural sensitivity, and commitment to ethical considerations position it as a valuable piece in the global mosaic of understanding mRNA vaccine safety [15]. As the world navigates through ongoing vaccination campaigns, the insights generated by this study carry weight for the broader global community facing challenges and concerns.

Strengths and weaknesses of the study

The study on self-reported physician-diagnosed cardiac complications post mRNA vaccination in Saudi Arabia exhibits several notable strengths. Its timely investigation into the rare but crucial aspect of cardiac complications aligns with global mRNA vaccination efforts. A comprehensive questionnaire, covering demographic details, health conditions, vaccination history, and perceptions, demonstrates methodological rigor. Ethical considerations, such as informed consent and confidentiality, underscore the commitment to participant well-being. However, the study faces limitations, including potential self-reporting bias and the use of convenience sampling, affecting generalizability. The cross-sectional design captures a single snapshot in time, and the accuracy of participants' vaccination history recall may vary. Despite these weaknesses, the study's strengths in methodology, relevance, and ethical considerations contribute valuable insights into the safety profile of mRNA vaccines in the specific context, fostering informed public health strategies in Saudi Arabia.

## Conclusions

The study on self-reported physician-diagnosed cardiac complications post mRNA vaccination in Saudi Arabia emerges as a crucial and timely contribution to the global discourse on vaccine safety. The advent of mRNA vaccines has marked a significant milestone in public health. This study, conducted in the distinctive demographic and epidemiological landscape of Saudi Arabia, not only recognizes the rarity of cardiac complications following vaccination but diligently investigates their incidence, nature, and associated factors. The study contributes to the global understanding of mRNA vaccine safety by providing real-world data that complements clinical trial findings. The insights generated by this research not only benefit the Saudi population but also contribute to the collective knowledge base on vaccine-related cardiac complications worldwide. As nations strive to build and maintain public trust in vaccination programs, especially with emerging vaccine technologies, studies of this nature play a pivotal role in fostering informed decision-making and ensuring the ongoing success and safety of vaccination campaigns.
